# A new variant of Abernethy malformation treated by transhepatic interventional closure: a case report

**DOI:** 10.1186/s12876-022-02123-1

**Published:** 2022-02-07

**Authors:** Ludger Sieverding, Michael Hofbeck, Jörg Michel, Andreas Hornung, Christian Scheckenbach, Gerd Grözinger, Ekkehard Sturm, Steven W. Warmann, Anja Hanser

**Affiliations:** 1 Department of Pediatric Cardiology, University Children’s Hospital, University of Tübingen, Hoppe-Seyler-Str. 1, 72076 Tübingen, Germany; 2 Department of Diagnostic and Interventional Radiology, University Hospital of Tübingen, Tübingen, Germany; 3 Department of Pediatric Gastroenterology and Hepatology, University Children’s Hospital, University of Tübingen, Tübingen, Germany; 4 Department of Pediatric Surgery and Pediatric Urology, University Children’s Hospital, University of Tübingen, Tübingen, Germany

**Keywords:** CPSS, Congenital portosystemic shunts, Abernethy malformation, Pulmonary hypertension, Transhepatic closure

## Abstract

**Background:**

Congenital portosystemic shunts (CPSS) are rare vascular malformations and can be classified into extrahepatic and intrahepatic shunts. Extrahepatic CPSS, also termed Abernethy malformations are associated with severe long-term complications including portopulmonary hypertension, liver atrophy, hyperammoniemia and hepatic encephalopathy. We report a hitherto undescribed variant of Abernethy malformation requiring an innovative approach for interventional treatment.

**Case presentation:**

We describe a 31-year-old patient following surgical repair of atrioventricular septal defect at the age of 6 years. In the long-term follow-up he showed persistent pulmonary hypertension which deteriorated despite dual pulmonary vasodilative treatment. When he developed arterial desaturation and symptomatic hyperammoniemia detailed reassessment revealed as underlying cause a hitherto undescribed variant of Abernethy malformation connecting the portal vein with the right lower pulmonary vein. Following interdisciplinary discussions we opted for an interventional approach. Since the malformation was un-accessible to interventional closure via antegrade venous or retrograde arterial access, a transhepatic percutaneous puncture of the portal vein was performed. Temporary balloon occlusion of the malformation revealed only a slight increase in portal venous pressure. Interventional occlusion of the large vascular connection was achieved via this transhepatic approach by successive implantation of two large vascular occluding devices. The postinterventional course was unremarkable and both ammonia levels and arterial saturation normalized at follow-up of 12 months.

**Conclusions:**

Portal vein anomalies should be included in the differential diagnoses of pulmonary hypertension or pulmonary arterio-venous malformations. Based on careful assessment of the anatomy and testing of portal vein hemodynamics interventional therapy of complex Abernethy malformations can be performed successfully in specialized centers.

## Background

CPSS are rare vascular malformations with an estimated incidence of one in 50,000 births [1]. Probably based on improvements of imaging techniques the number of reported cases has increased significantly during the recent years [2]. Development of CPSS is attributed to failed involution of one or several primordial venous vessels resulting in abnormal vascular connections between portal and systemic veins. These abnormal vascular connections allow partial or complete bypass of portal venous flow from the liver to the systemic veins [1, 3]. Early complications of CPSS include neonatal cholestasis and galactosemia as a consequence of portosystemic shunting. Late complications encompass liver atrophy which is probably related to portal venous flow bypassing the liver and absent hepatotrophic factors [2, 4, 5]. Other liver pathologies include focal nodular hypoplasia as well as development of benign or malignant liver tumors like hepatocellular adenoma and hepatocellular carcinoma [1, 2, 6]. Screening tests for early detection of hepatic tumours should be carried out regularly in the follow-up of these patients. Significant morbidity and mortality may result from portopulmonary hypertension and hepatopulmonary syndrome. Pulmonary hypertension may occur both in neonates and in adults. Franchi-Abella et al. reported portopulmonary hypertension in 40 of 413 patients, 31 of these patients having extrahepatic shunts. In at least six patients, sudden cardiac death was associated with pulmonary hypertension [2]. Hepatopulmonary syndrome with the development of intrapulmonary arteriovenous-shunts results in chronic hypoxemia. Portosystemic encephalopathy often associated with increased ammonemia is the most frequent neurologic complication which may develop at any age [1, 2, 6].

John Abernethy was the first to describe absence of the portal vein associated with an extrahepatic CPSS in 1793 [7]. Since then several different classifications have been introduced to classify these abnormal vascular connections. CPPS can be classified in an extrahepatic and an intrahepatic category. Extrahepatic shunts are classified in type 1 characterized by total congenital absence of the portal vein, and type 2 associated with partial congenital absence of the portal vein [8]. Both anomalies are referred to as Abernethy malformations [2, 8, 9]. Intrahepatic shunts are located inside the liver. Based on a large cohort of 265 children Bernard et al. suggested a different classification based on the origin of the CPSS from the portal system, the type and number of communications [1]. Finally, the classification proposed by Franchi-Abella [2] is based on surgical and interventional treatment options according to Blanc [10] and Kanazawa [4]. Congenital CPSS can be distinguished from acquired CPSS which are described in up to 20% of patients with increasing portal hypertension in the context of liver cirrhosis. These acquired vascular anastomoses are characterized by a varicose appearance [11].

While CPSS connecting to the right atrium have been described in two children of the cohort of Bernard et al. [1] there are no cases of abnormal shunts reported in the literature connecting the portal vein system to pulmonary veins. We report a hitherto undescribed variant of Abernethy malformation requiring an innovative approach for interventional treatment [1, 2, 12].

## Case presentation

### Preinterventional history and evaluation

**Fig. 1:**
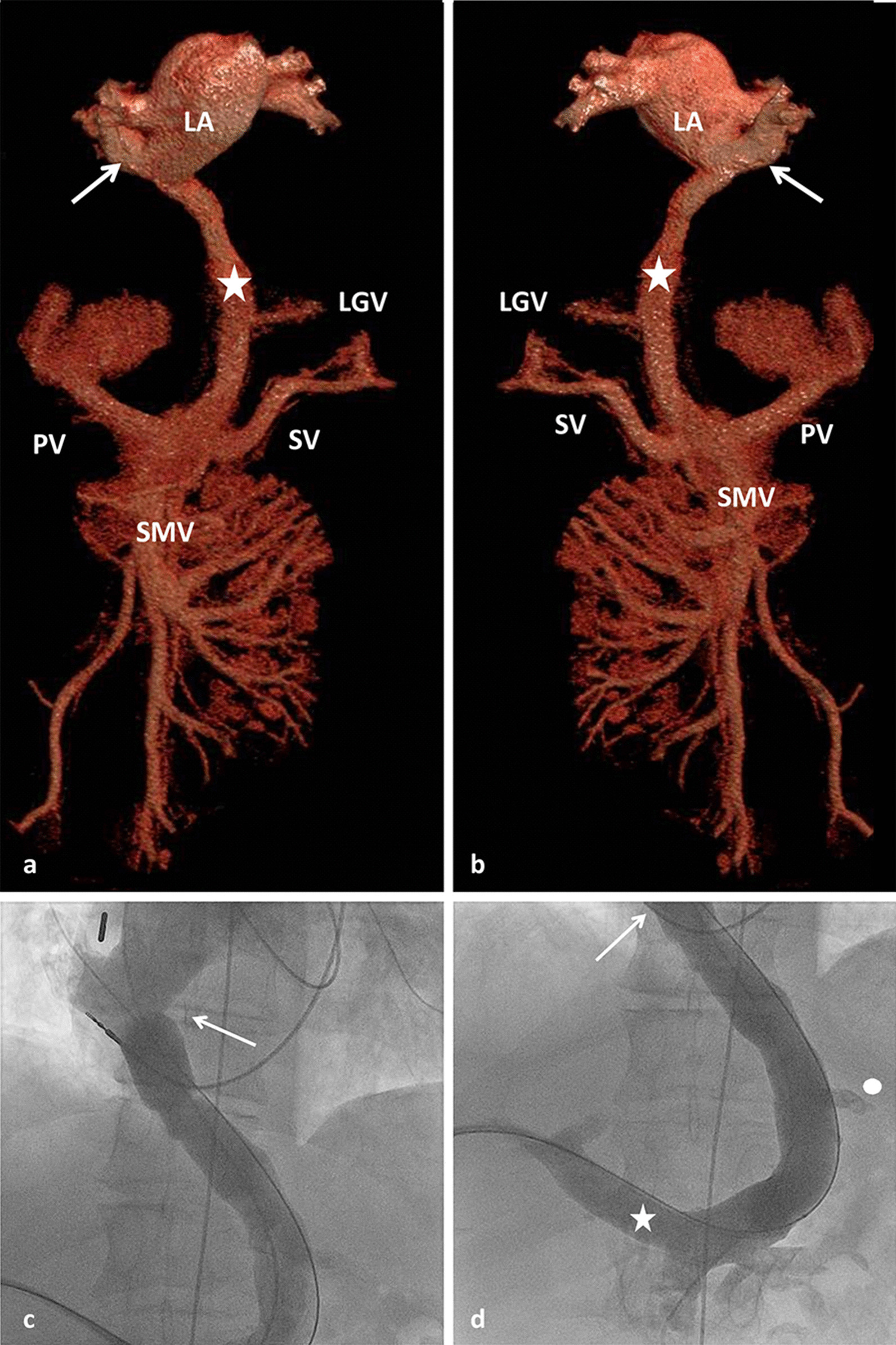
**a** CT reconstruction (ap-view) showing the shunt vein (star) connecting the portal vein (PV) to the right lower pulmonary vein (arrow). SV: splenic vein, SMV: superior mesenteric vein, LGV: left gastric vein, LA: left atrium. **b** CT reconstruction (pa-view) showing the shunt vein (star) connecting the portal vein (PV) to the right lower pulmonary vein (arrow). SV: splenic vein, SMV: superior mesenteric vein; LGV: left gastric vein, LA: left atrium. **c** Angiography confirming the anastomosis (arrow) of the shunt vein to the right lower pulmonary vein, which opens into the left atrium. **d** Transhepatic access of the portopulmonary connection. Angiography showing the shunt vein extending from the portal vein (star) toward the inferior pulmonary vein (arrow); left gastric vein (circle)

**Fig. 2:**
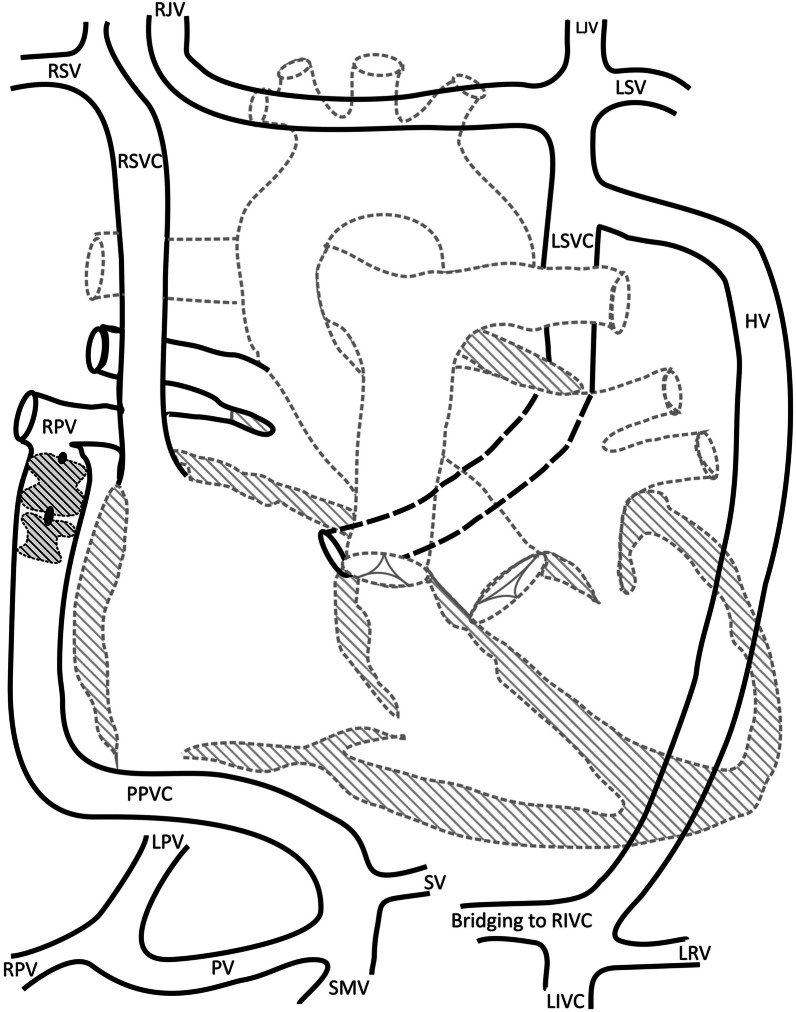
Schematic representation of the venous vessels: RSV: right subclavian vein; RJV: right internal jugular vein; LJV: left internal jugular vein; LSV: left subclavian vein; RSVC: right superior vena cava; LSVC: left superior vena cava; HV: hemazygos vein; RPV: right pulmonary vein; PPVC: portopulmonary venous connection; SV: splenic vein; SMV: superior mesenteric vein; PV: portal vein; LPV: left portal vein; RPV. Right portal vein; RIVC: right inferior vena cava (suprarenal); LIVC: left inferior vena cava; LRV: left renal vein

We report a 31-year-old male patient who presented in our center for treatment of a complex cardiovascular malformation. Detailed evaluation of the history revealed that at the age of 6 7/12 years the patient had corrective surgery for atrioventricular septal defect (AVSD) and pulmonary stenosis. Pulmonary hypertension was present at that time despite the severely stenotic pulmonary valve. Repeat cardiac catheterization at the age of 21 years revealed pulmonary hypertension with a mean pulmonary arterial pressure of 32 mmHg and elevated pulmonary vascular resistance index (6.4 WE*m^2^). The systemic oxygen saturation was 98%. At that time he also required pacemaker implantation for treatment of symptomatic bradycardia caused by AV block type Mobitz II. A CT-scan performed prior to this procedure revealed a large anomalous vessel, connecting the portal vein to the right lower pulmonary vein (Fig. 1a–d). In addition the patient presented complex anomalies of the systemic veins suggesting disturbed embryonic lateralisation of the venous system: 1. Bilateral superior vena cava with drainage of the left superior vena cava into the coronary sinus; 2. Isolated drainage of the right internal jugular vein into the left superior vena cava via a brachiocephalic vein; 3. Interrupted left inferior vena cava with hemiazygos continuation to the left superior vena cava and a bridging vein to a suprarenal right inferior vena cava (Fig. 2). Further, the liver parenchyma was inhomogenous and showed an inhomogenous nodular pattern.

Oral pulmonary vasodilative therapy was initiated with the PDE5 inhibitor sildenafil and extended by the addition of the endothelin receptor antagonist macitentan when systemic saturation dropped to 90%. Since hemodynamics remained unchanged despite dual pulmonary vasodilatative treatment the patient was admitted in our center. Reevaluation of the complex venous anatomy brought to our attention the previously described porto-pulmonary venous connection (PPVC). In this context we detected hyperammonemia with 103 µmol/l. Because of the combination of pulmonary hypertension, arterial desaturation and symptomatic hyperammoniaemia with impaired concentration we performed interdisciplinary discussion of the case and opted for an occlusion of the PPVC by an endovascular approach.

### Invasive testing and Intervention

The intervention was performed under general anesthesia and full heparinization. To allow pressure measurements and angiography of the portal vein we started with retrograde catheterization of the portal vein from the right femoral artery via the left ventricle, left atrium, right inferior pulmonary vein and the abnormal PPVC. Since the shunt vessel was inaccessible for retrograde placement of a large sheath required for interventional occlusion we decided to perform the interventional occlusion via a transhepatic approach. Percutaneous transhepatic puncture of the portal vein was performed under sonographic guidance followed by insertion of a 4 French sheath. This access was subsequently dilated up to a 9 French sheath. Via this access we probed the PPVC and inserted a 0.035" extra stiff wire into the right lower pulmonary vein. Over this wire a 25 mm sizing balloon was inserted to perform temporary balloon occlusion of the abnormal vessel. The balloon diameter required for complete occlusion of the PPVC was 19 mm. Pressure measurements performed with the retrograde catheter inserted from the femoral artery revealed almost no change in mean portal venous pressure which increased under occlusion of the PPVC from 17 up to a maximum of 20 mmHg confirming the possibility of interventional occlusion of this abnormal vessel without subsequent portal hypertension.

**Fig. 3:**
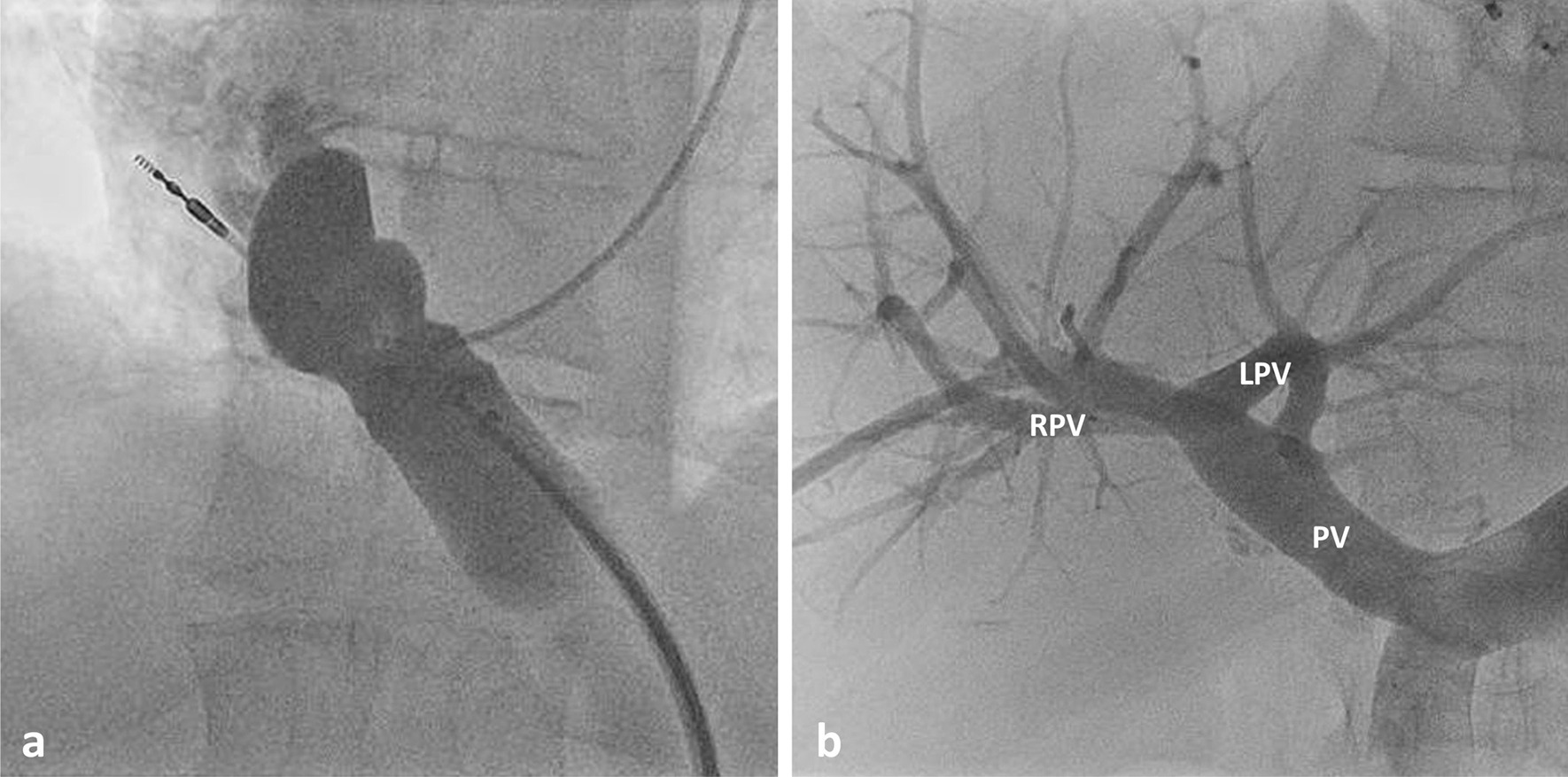
**a** Contrast agent administration into the shunt vein directly after insertion of the two occluders. **b** Contrast staining of the intrahepatic portal veins at the end of the procedure. PV: portal vein; LPV: left portal vein; RPV: right portal vein

Based on this information we decided to close the PPVC. The retrograde arterial catheter was withdrawn and an 8 French sheath was inserted and advanced into the PPVC up to its distal end at the right pulmonary vein. Via this sheath two large occluding devices (18 mm Amplatzer™ Septal Occluder and 16 mm Amplatzer™ Duct Occluder; Abbott Medical, Plymouth, MN, USA) were implanted successively into the PPVC (Fig. 3a).

## Results

Contrast medium injection into the portal vein at the end of the procedure showed an almost immediate complete occlusion of the CPSS and drainage of the contrast medium via the right and left intrahepatic portal vein (Fig. 3b). Subsequently the sheath was retracted into the liver parenchyma and a vascular plug (8 mm Amplatzer™ Vascular Plug 4; Abbott Medical, Plymouth, MN, USA) was implanted into the branch canal followed by filling the branch canal with a haemostatic absorbable gelatine sponge.

The postinterventional course was unremarkable. Oral anticoagulation was started with phenprocumon to prevent possible thrombosis of the portal vein. At follow-up 12 months later the patient is doing well. Both systemic saturation (97%) and ammonia level (32 mmol/l) returned to normal (97%) while sonography revealed hepatopetal portal vein flow and signs of liver remodeling. However repeat echocardiography 15 months after intervention revealed tricuspid regurgitation with a systolic gradient of 38 mmHg. This finding reflects persisting pulmonary hypertension despite successful closure of the CPSS and despite dual pulmonary vasodilative therapy.

## Discussion and conclusions

There is consensus that CPSS should be treated in symptomatic patients. According to the data of the International Registry of Congenital Portosystemic Shunts even pronounced hepatic pathologies are reversible after successful CPSS occlusion [12]. Reestablishment of intrahepatic portal flow may also lead to complete regression of benign and even malignant liver tumors. Although it is less clear whether CPSS should be closed in asymptomatic patients there are some good arguments for prophylactic treatment including the prevention of possible damage to the liver, hepatopulmonary hypertension, hepatopulmonary syndrome and portosystemic encephalopathy [1, 6, 12, 13]. Since oxygen saturation gradually declined and pulmonary hypertension progressed in our patient despite dual pulmonary vasodilative therapy and as he developed increasing disturbance of concentration in the presence of hyperammonaemia we saw a clear indication for occlusion of the vascular malformation. Although a hepatopulmonary syndrome could not be excluded with certainty, desaturation in our patient was most likely due to shunt flow to the right pulmonary vein resulting in desaturation of the LA (85%) in the presence of normal saturations in the right upper and left pulmonary veins. In contrast, the combination of pulmonary hypertension and hepatopulmonary syndrome is rare: Bernard et al. in their review of 265 children reported the association between pulmonary hypertension and hepatopulmonary syndrome in 5 children only [1].

Detailed assessment of the anatomy and hemodynamics of CPSS is essential to prepare interventional or surgical occlusion. Angiographic imaging of the extra- and intrahepatic portal vein during temporary occlusion of the CPSS is strongly advised prior to the treatment of all variations of Abernethy malformations. The detection of even extremely hypoplastic portal vein branches opens the option of partial or complete closure of the abnormal vessel. Decisive for the planning of surgical or interventional procedures is the increase in pressure within the portal vein system under temporary balloon occlusion. A cut-off value of 32 mmHg under occlusion conditions has been specified by the Paris working group [1, 12]. In patients with a portal venous pressure below 32 mmHg the CPSS can be closed safely in a one stage procedure.

During the recent years interventional occlusion of CPSS has become increasingly important. This can be achieved either by one stage intravascular embolization procedures using vascular plugs [14, 15], coils [16, 17] or even due to the considerable and variable size of the shunt vessels ASD-, VSD-, or PDA-Occluders [1, 12]. Another option are two stage procedures aiming to achieve reduction of the diameter of the CPSS by surgical banding or by implantation of a restrictive stent followed later by complete occlusion of the shunt vessel [18]. The latter technique is preferred to allow adaptive growth in the presence of a severely hypoplastic intrahepatic portal vein system [12, 13, 18]. Since the measurements carried out in our patient showed a maximum portal vein pressure of 20 mmHg under occlusion conditions and since the intrahepatic portal vein branches were of approximately normal size we decided to perform primary complete closure of the abnormal vessel.

To the best of our knowledge this is the first case in which closure of a congenital CPSS has been performed via a percutaneous transhepatic access. This approach was required in our patient since the portal vein could not be reached easily from the systemic veins and a retrograde approach via aorta, left ventricle and left atrium was impossible for placement of the required large size long sheath.

A transjugular approach with subsequent transhepatic puncture of the portal vein would have been a conceivable alternative, but it would have required a more complex guidance and route of the catheter. Despite the more direct access associated with the chosen percutaneous transhepatic approach, placement of the large-size sheath was still very difficult: Advancement of the sheath required insertion of an extra-stiff guide wire and creation of an abutment by inflation of a balloon catheter in the shunt vein. Another possibility could have been a venous access followed by transseptal puncture of the left atrium with subsequent probing of the CPSS form the right lower pulmonary vein. This approach however would have required a much more complex guidance of the catheter and long sheath.

In contrast to the alternative of surgical treatment the intervention provided the possibility to evaluate the portal venous pressure under temporary occlusion of the abnormal vessel. Due to the large size of the PPVC safe occlusion required the successive placement of two large devices designed for occlusion of atrial septal defects and persistent ductus arteriosus. Surgical treatment is usually required for the treatment of extrahepatic shunts with short length [1]. Through the advances in interventional and surgical closures, liver transplantation has become very rare in patients with CPSS [1, 12].

In conclusion portal vein anomalies should be included in the differential diagnoses of pulmonary hypertension or pulmonary arterio-venous malformations. Upon identification of CPSS in symptomatic patients, the vascular malformation should be evaluated for the suitability for interventional occlusion. Occlusion procedures require careful assessment of the anatomy and testing of the portal vein hemodynamics before occlusion. Since interventional therapy may require unusual vascular access and large occluding devices and due to the complex nature of these rare lesions interdisciplinary therapy should be concentrated in centers with specific expertise in these malformations.

## Data Availability

The datasets used and/ or analysed during the current study are available from the corresponding author on reasonable request.

## Abbreviations

ASD: Atrial septal defect
AVSD: Artrioventricular septal defect
AV block: Atrioventricular block
BSA: Body surface area
IVC: Inferior vena cava
PAPm: Mean pulmonary arterial pressure
PAPVC: Partial anomalous pulmonary vein connection
PDA: Patent ductus arteriosus
PM: Pacemaker
LA: Left atrium
PPVC: Portopulmonary venous connection
RPI: Pulmonary vascular resistance index
VSD: Ventricular septal defect
